# Anti-Tumor Activity of Stevia Leaf Extract Fermented by the Plant-Derived *Lactiplantibacillus plantarum* SN13T in a Pancreatic Tumor Xenograft Model

**DOI:** 10.3390/antiox15050581

**Published:** 2026-05-04

**Authors:** Rentao Zhang, Narandalai Danshiitsoodol, Masafumi Noda, Masanori Sugiyama

**Affiliations:** 1 Department of Probiotic Science for Preventive Medicine, Graduate School of Biomedical and Health Sciences, Hiroshima University, Hiroshima 734-8551, Japan; d225530@hiroshima-u.ac.jp (R.Z.); bel@hiroshima-u.ac.jp (M.N.); 2 Gansu Province Key Laboratory of Environmental Oncology, Lanzhou University Second Hospital, Lanzhou 730000, China

**Keywords:** antioxidant, apoptosis, *Lactiplantibacillus plantarum*, pancreatic cancer, stevia

## Abstract

Background: Pancreatic cancer is a highly aggressive malignancy with a poor prognosis and limited efficacy of conventional therapies. Developing safe and effective natural adjuvant therapies is therefore of considerable interest. This study evaluated the in vivo anti-tumor activity of stevia leaf extract fermented by *Lactiplantibacillus* (*L.*) *plantarum* SN13T in an ectopic PANC-1 xenograft mouse model and explored potential mechanisms associated with its observed biological effects. Methods: Mice with PANC-1 tumors were randomly assigned to four groups: normal saline (NC group), unfermented stevia leaf extract (SLE, Unfer group), fermented stevia leaf extract by *L. plantarum* SN13T (FSLE, Fer group), and capecitabine (PC group). Body weight and tumor volume were monitored throughout the experiment. At the study termination, serum, tumor, liver, and kidney samples were collected for biochemical assays, hematoxylin–eosin and immunohistochemical staining, cytokine measurements, Western blot and qPCR analyses, and antioxidant measurements. Results: Fermented stevia leaf extract significantly inhibited tumor growth, as evidenced by reduced tumor volume and weight. Serum pro-inflammatory cytokines (IL-6, TNF-α, and IL-1β) were markedly decreased, accompanied by improvement in liver injury markers (ALT, AST) and lactate dehydrogenase (LDH). In tumor tissues, FSLE was associated with increased protein expression of cleaved caspase-3 and Bax, along with decreased Bcl-2 levels. Notably, Nrf2 protein expression in tumor tissues was downregulated, while intratumoral IL-6 levels were also decreased. In the liver, treatment was accompanied by increased Nrf2 and HO-1 mRNA expression, enhanced superoxide dismutase (SOD) activity, and reduced malondialdehyde (MDA) levels. Conclusions: FSLE exerts anti-tumor effects in a PANC-1 xenograft model that are associated with the modulation of inflammation, oxidative stress, and apoptosis-related pathways. These observations provide experimental and theoretical support for the further development of fermented plant-derived products as adjunctive therapies for pancreatic cancer.

## 1. Introduction

Pancreatic cancer is a highly aggressive malignancy characterized by poor prognosis [1]. Its early-stage symptoms are often nonspecific and easily overlooked, and most patients are diagnosed at advanced stages when surgical resection is not feasible. Epidemiological data indicate a 5-year survival rate below 10% and high mortality, making pancreatic cancer a leading cause of cancer-related deaths worldwide [2]. Current therapeutic modalities, including radiotherapy, chemotherapy, and targeted therapies, provide limited clinical benefit. They are often associated with relapse, drug resistance, and considerable adverse effects [3,4]. Thus, developing novel therapeutic strategies that are safe, effective, and low in toxicity remains a critical priority.

Natural products have garnered significant interest as adjuvants in cancer therapy due to their abundance, multi-target pharmacological effects, and relatively low toxicity [5]. These compounds exhibit antioxidant, anti-inflammatory, and immunomodulatory activities and can suppress tumor growth by inducing apoptosis and inhibiting cell proliferation and migration [6]. Stevia (*Stevia rebaudiana* Bertoni), widely used as a natural sweetener, contains leaf extracts rich in steviol glycosides, flavonoids, and polyphenols [7]. Emerging evidence indicates that stevia leaf extracts possess antioxidant, anti-inflammatory, and potential anti-cancer properties, with certain constituents modulating cell cycle and apoptosis pathways to inhibit tumor growth [8,9,10]. However, the clinical application of stevia-derived compounds is limited by low bioavailability and metabolic instability.

Microbial fermentation represents a promising approach to enhancing the pharmacological properties of natural products. Lactic acid bacteria (LAB), classified as Generally Recognized As Safe (GRAS) [11], are widely used to biotransform plant extracts via esterification, glycosylation, and small-molecule degradation, thereby generating derivatives with improved activity [12,13,14]. Fermentation can increase component stability and solubility, boost bioavailability, and expand biological targets. Fermentation by LAB strains has been shown to enhance the antiproliferative and pro-apoptotic effects of medicinal plants such as barley and ginseng. Certain fermentation products can also modulate the immune microenvironment to support anti-tumor responses [15,16]. Notably, the fermentation of cinnamon by *Lactiplantibacillus (L.) plantarum* has been reported to enhance its anti-cancer potential without generating toxic metabolites [17], underscoring the safety and efficacy advantages of this approach.

Our previous in vitro work demonstrated that *L. plantarum* SN13T-mediated fermentation of stevia leaf extract markedly inhibited PANC-1 pancreatic cancer cell proliferation and migration and effectively induced apoptosis [18]. Based on these findings, the present study employed an ectopic PANC-1 xenograft nude mouse model to evaluate the in vivo anti-tumor activity of *L. plantarum* SN13T-fermented stevia leaf extract (FSLE), and extend our prior findings while providing preliminary observations related to potential mechanisms underlying its biological effects. These data offer initial experimental support for the potential application of FSLE as an adjunct strategy for pancreatic cancer management.

## 2. Materials and Methods

### 2.1. Preparation of FSLE

Extraction and preparation procedures were performed as previously described [18]. Briefly, *L. plantarum* SN13T was activated and cultured in de Man, Rogosa, and Sharpe (MRS) broth (Merck KGaA, Darmstadt, Germany) at 37 °C for 24 h under anaerobic conditions. Bacterial cells were collected by centrifugation and resuspended in an equal volume of sterile phosphate-buffered saline (PBS). Dried stevia leaves (5 g, Kojima Kampo Co., Ltd., Osaka, Japan) were soaked in 100 mL of distilled water and heated at 105 °C for 30 min. The mixture was cooled, centrifuged, and the supernatant was filtered to obtain the sterile stevia leaf extract. The *L. plantarum* SN13T suspension was inoculated into the stevia leaf extract at 1% (*v*/*v*) and incubated anaerobically at 37 °C for 72 h. After fermentation, the culture was centrifuged and filtered, and the supernatant was collected as FSLE.

### 2.2. Cell Culture

The human pancreatic cancer cell line PANC-1 (RBRC, RCB2095) was obtained from the RIKEN BRC Cell Bank. The cells were cultured in Roswell Park Memorial Institute (RPMI1640) medium supplemented with 10% fetal bovine serum (FBS, Life Technologies Co., Grand Island, NY, USA) and 1% penicillin/streptomycin solution (FUJIFILM Wako Pure Chemical Co., Osaka, Japan) at 37 °C in a humidified incubator with 5% CO_2_.

### 2.3. Establishment of an Ectopic Pancreatic Cancer Model

All animal procedures were approved by the Hiroshima University Animal Experimentation Ethics Committee (Protocol code A24-101) and were performed in accordance with the Hiroshima University Guidelines for the Care and Use of Laboratory Animals. Five-week-old male BALB/c-nu/nu mice (SPF grade) were purchased from Shimizu Laboratory Supplies Co., Ltd. (Kyoto, Japan) and maintained under controlled conditions (20–26 °C, 40–60% relative humidity, 12 h light/dark cycle) with free access to food and water throughout the study.

After acclimatization, 2 × 10^6^ PANC-1 cells were mixed 1:1 (*v*/*v*) with cold Matrigel (100 μL; Corning Inc., New York, NY, USA) and injected subcutaneously into the right flank of each mouse. Body weight was monitored from the day of tumor inoculation (Day 7) and continued for 40 days, whereas tumor volume was measured from the first day of treatment (Day 13 post-inoculation) for a total of 34 days. Tumor volume (V) was calculated using the following formula:

Vmm3=12(L×W2)

where L is the longest diameter and W is the diameter perpendicular to L.

When tumor volume reached approximately 100 mm^3^, mice were randomized into four groups (*n* = 4 per group): normal saline (NC group), unfermented stevia leaf extract (SLE, Unfer group), FSLE (Fer group), and capecitabine (PC group, 30 mg/mL). Test articles were administered by oral gavage (200 μL per dose) once daily. The daily oral dose (200 μL per mouse) of FSLE (36.2 mg/mL) and SLE (37.8 mg/mL) was selected based on the effective and well-tolerated dose range established in our previous study [18,19]. Tumor length and width were measured with calipers every 3 days, and body weight was recorded. Tumor growth curves were plotted based on calculated volumes. At the study endpoint, tumors were excised and weighed; tumor growth inhibition was calculated as:

$$Inhibition\;(\%)=\left(m_{1}-m_{2}\right)/m_{1}\times 100$$

where $m_{1}$ and $m_{2}$ represent the mean tumor weights of the NC group and the treatment group, respectively.

At the study endpoint, mice were euthanized under isoflurane anesthesia. Blood was collected via the abdominal aorta, and serum was separated by centrifugation at 4000× *g* for 10 min at 4 °C and stored at −80 °C. Liver, kidney, and tumor tissues were fixed in 4% paraformaldehyde for histological analysis or snap-frozen in liquid nitrogen and stored at −80 °C for molecular analyses.

### 2.4. Serum Biochemical and Cytokine Assays

Serum levels of aspartate aminotransferase (AST), alanine aminotransferase (ALT), alkaline phosphatase (ALP), lactate dehydrogenase (LDH), cholinesterase (ChE), blood urea nitrogen (BUN), and uric acid (UA) were measured using commercial assays provided by Oriental Yeast Co., Ltd. (Tokyo, Japan). Serum cytokines, including interferon-γ (IFN-γ; Cat: 430801, BioLegend, San Diego, CA, USA), interleukin-6 (IL-6; Cat: 431301, BioLegend, San Diego, CA, USA), tumor necrosis factor-α (TNF-α; Cat: 430901, BioLegend, San Diego, CA, USA), and interleukin-1β (IL-1β; Cat: 432601, BioLegend, San Diego, CA, USA), were quantified using commercial ELISA kits according to the manufacturers’ instructions. In addition, IL-6 and TNF-α levels in tumor tissue homogenates were also measured using the corresponding ELISA kits to evaluate local inflammatory responses. Total protein concentrations in tumor homogenates were measured by the bicinchoninic acid (BCA) method using bovine serum albumin (BSA) as the standard, and cytokine levels were normalized to protein content. Absorbance at 450 nm was measured with a microplate reader, and cytokine concentrations were calculated from standard curves.

### 2.5. Assessment of Hepatic Oxidative Stress Markers

Liver tissues were homogenized in a sucrose buffer and PBS containing antioxidants (10 mg tissue per 100 μL buffer). Homogenates were centrifuged at 10,000× *g* for 60 min at 4 °C, and the resulting supernatants were collected for assays of superoxide dismutase (SOD) activity and malondialdehyde (MDA) content. SOD activity was measured using the SOD Assay Kit-WST (Code: S311, Dojindo, Kumamoto, Japan), and MDA levels were measured using the MDA Assay Kit (Code: M496, Dojindo, Kumamoto, Japan). Absorbance was recorded with a microplate reader (Varioskan, Thermo Scientific, Vantaa, Finland) following the manufacturer’s instructions. Total protein concentrations were determined as described above, and SOD and MDA values were normalized to protein content. SOD activity is reported as U/mg protein, and MDA content as nmol/mg protein.

### 2.6. Liver Tissues RNA Extraction and qRT-PCR Analysis

Total RNA was extracted from mouse liver tissues using the NucleoSpin RNA Plus Kit (Macherey-Nagel, Duren, Germany), and reverse transcribed with ReverTra Ace qPCR RT Master Mix with gDNA Remover (Toyobo, Osaka, Japan). qRT-PCR was carried out with the KAPA SYBR Fast qPCR Kit (Kapa Biosystems, Woburn, MA, USA) on a PikoReal Real-Time PCR System (Thermo Fisher Scientific, Waltham, MA, USA).

The qPCR conditions were as follows: 95 °C for 2 min, followed by 40 cycles of 95 °C for 5 s and 60 °C for 10 s. Relative mRNA expression levels were normalized to GAPDH and analyzed using the ΔΔCT method. The primer sequences were as follows: GAPDH: F, 5′-CATCACTGCCACCCAGAAGACTG-3′; R, 5′-ATGCCAGTGAGCTTCCCGTTCAG-3′; Nrf2: F, 5′-CAGCATAGAGCAGGACATGGAG-3′; R, 5′-GAACAGCGGTAGTATCAGCCAG-3′; HO-1: F, 5′-CACTCTGGAGATGACACCTGAG-3′; R, 5′-GTGTTCCTCTGTCAGCATCACC-3′.

### 2.7. Western Blot Analysis

Total protein was extracted from mouse tumor tissues using a cell lysis buffer M (Cat: 038-21141, FUJIFILM Wako, Japan) containing 1% phosphatase inhibitor cocktail, and protein concentrations were determined by the BCA method. Equal amounts of protein were resolved by 10% SDS-PAGE and electrotransferred onto polyvinylidene difluoride (PVDF) membranes (EMD Millipore, Burlington, MA, USA). Membranes were blocked with 5% nonfat milk at room temperature for 1 h. These were then incubated overnight at 4 °C with the following primary antibodies: Bax (1:20,000, Cat: 50599-2-IG, Cosmo Bio Co., Ltd., Tokyo, Japan), Bcl-2 (1:5000, Cat: 12789-1-AP, Cosmo Bio Co., Ltd., Tokyo, Japan), cleaved caspase-3 (1:10,000, Cat: 68773-1-IG, Cosmo Bio Co., Ltd., Tokyo, Japan), Nrf2 (1:10,000, Cat: 66504-1-IG, Cosmo Bio Co., Ltd., Tokyo, Japan), and β-actin (1:10,000, Cat: 6609-1-IG, Cosmo Bio Co., Ltd., Tokyo, Japan). After washing with TBST, membranes were incubated with HRP-conjugated goat anti-rabbit secondary antibody (1:5000, GeneTex Inc., Irvine, CA, USA) at room temperature for 1 h. Protein bands were visualized using the ImmunoStar LD Kit (Cat: 296-69901; FUJIFILM Wako Pure Chemical Co., Osaka, Japan) and quantified by densitometry with ImageJ software (version 1.52, National Institutes of Health, Bethesda, MD, USA).

### 2.8. Histological Analysis

Liver, kidney, and tumor tissues were fixed in 4% paraformaldehyde, dehydrated through a graded ethanol series, embedded in paraffin, and sectioned at 4–5 µm thickness for histological analysis. The sections were dried at 60 °C, cooled, deparaffinized, rehydrated, stained with hematoxylin and eosin, dehydrated, cleared, and mounted with neutral mounting medium. Histopathological changes in the liver, kidney, and tumor tissues of each group were examined under an Olympus IX71 microscope (Olympus Corp., Tokyo, Japan).

### 2.9. Immunohistochemical (IHC) Analysis

Tumor tissues were fixed in 4% paraformaldehyde, embedded in paraffin, and sectioned at 4 µm. After deparaffinization and rehydration, sections were treated with 1× Tris-EDTA buffer (pH 9.0) for antigen retrieval. Endogenous peroxidase activity was blocked with 3% H_2_O_2_, followed by incubation with 5% goat serum. Sections were incubated overnight at 4 °C with primary antibodies against cleaved caspase-3. After washing, they were incubated with HRP-conjugated secondary antibody (Cat: PK10010, Cosmo Bio Co., Ltd., Tokyo, Japan). Immunoreactivity was visualized using DAB substrate and counterstained with hematoxylin. Images were captured using a microscope. Positive staining was quantified in ImageJ by measuring the integrated optical density (IOD) of the positively stained areas.

### 2.10. Statistical Analysis

All data are presented as the mean ± standard deviation (SD). Statistical analyses were performed using SPSS 22.0 (IBM Corp., Armonk, NY, USA) and GraphPad Prism 9.0 (San Diego, CA, USA). Differences among multiple groups were evaluated by one-way analysis of variance (ANOVA) followed by Tukey’s post hoc test. Statistical significance was expressed as * *p* < 0.05, ** *p* < 0.01, and *** *p* < 0.001.

## 3. Results

### 3.1. FSLE Significantly Inhibits Tumor Growth in Mice

**Figure 1:**
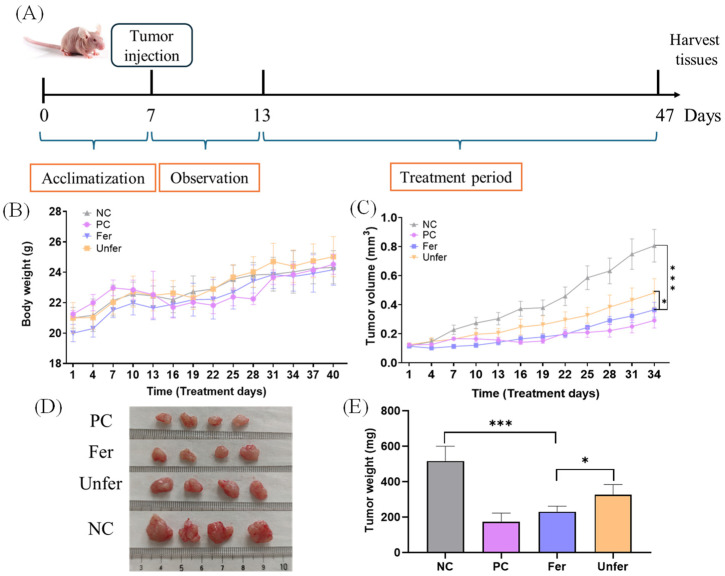
Inhibitory effect of FSLE on pancreatic tumor growth in mice. (**A**) Schematic diagram of the experimental design. (**B**) Body weight changes of mice in each group. (**C**) Tumor volume changes of mice in each group. (**D**) Representative images of tumors after sacrifice. (**E**) Tumor weights of mice in each group. Data are shown as mean ± SD. * *p* < 0.05, and *** *p* < 0.001.

To evaluate the in vivo anti-tumor activity of FSLE, the PANC-1 xenograft mouse model was established, and mice were orally administered the treatments once daily for 34 consecutive days (Figure 1A). Tumor growth was monitored throughout treatment. As shown in Figure 1B, body weights in all groups increased progressively over the course of the study, with no observable signs of treatment-related toxicity. Figure 1C illustrates that starting from day 7 post-inoculation, tumors in the NC group grew rapidly. In contrast, tumor growth in the Fer group was markedly attenuated. At the end of the experiment, the mean tumor volume in the NC group reached 806 ± 113 mm^3^, whereas the Fer group exhibited a significant reduction to 361 ± 52 mm^3^, corresponding to a tumor inhibition rate of 55.21%. In comparison, the Unfer group showed only modest tumor suppression (491 ± 88 mm^3^; inhibition rate 39.11%). The PC group treated with capecitabine displayed the smallest tumor volume (292 ± 52 mm^3^; inhibition rate 63.77%).

Representative images of excised tumors (Figure 1D) confirmed the differences in tumor size among the groups. Quantification of tumor weights (Figure 1E) corroborated the tumor volume measurements. The NC group averaged 566 ± 84 mg, whereas the Fer group was significantly reduced to 267 ± 48 mg. The Unfer group had a tumor weight of 351 ± 71 mg, and the PC group averaged 198 ± 45 mg.

### 3.2. FSLE Improves Hepatorenal and Antioxidant Function

#### 3.2.1. Effects of FSLE on Hepatorenal Function Parameters

**Figure 2:**
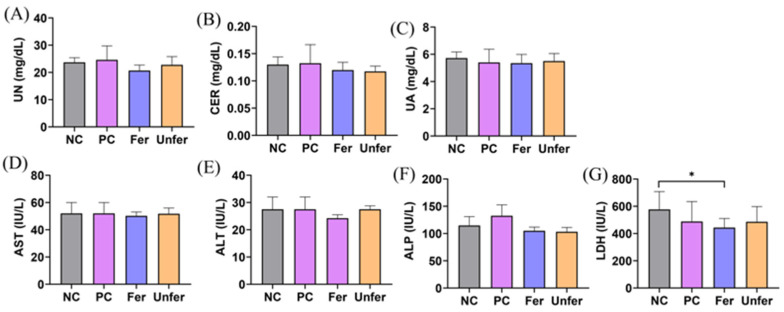
Effects of FSLE on serum biochemical parameters in mice. Liver and kidney function: serum biochemical parameters (**A**) BUN, (**B**) CRE, (**C**) UA, (**D**) AST, (**E**) ALT, (**F**) ALP, and (**G**) LDH. Data are shown as mean ± SD. * *p* < 0.05.

To evaluate the effects of FSLE on liver and kidney function in tumor-bearing mice, serum levels of renal function parameters (BUN, CRE, UA) and liver function parameters (AST, ALT, ALP, and LDH) were measured (Figure 2). BUN, CRE, and UA levels in the Fer group were similar to those in the NC group. Liver function parameters, including AST, ALT, and ALP, also showed no notable elevation compared with the NC group. Notably, LDH levels in the NC group were 577.0 ± 116.1 IU/L, whereas the Fer group showed a reduction to 443.8 ± 67.7 IU/L. A comparable but weaker decreasing trend was observed in the Unfer group.

#### 3.2.2. FSLE Alleviates Oxidative Stress in Liver

**Figure 3:**
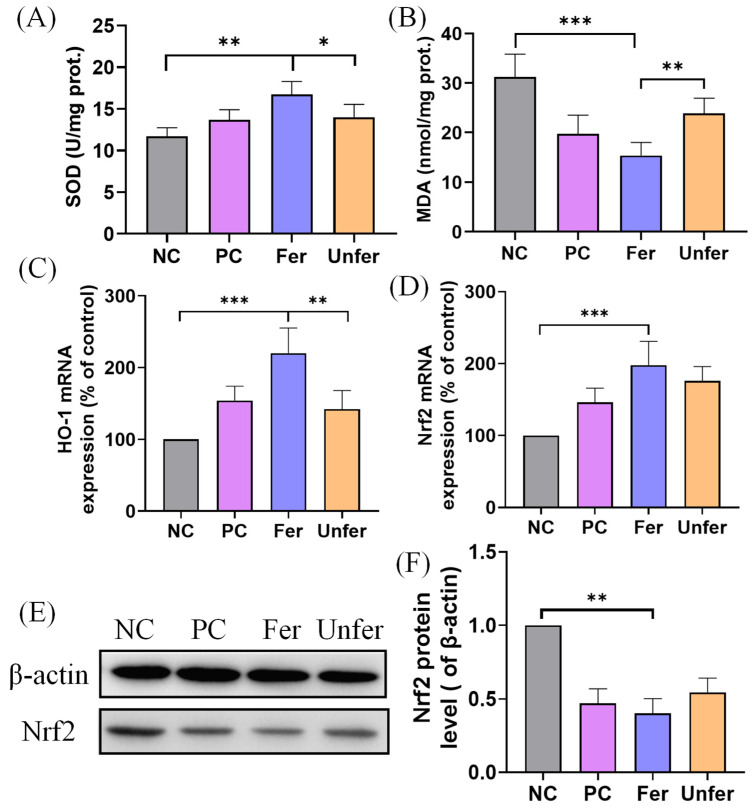
Effects of FSLE on antioxidant parameters in mice. (**A**) SOD activity in liver tissues. (**B**) MDA content in liver tissues. (**C**) HO-1 mRNA expression levels in liver tissues. (**D**) Nrf2 mRNA expression levels in mouse liver tissues. (**E**) Western blot analysis of Nrf2 protein expression in tumor tissues. (**F**) Bar graphs showing relative protein levels of Nrf2 normalized to β-actin. Data are presented as mean ± SD. * *p* < 0.05, ** *p* < 0.01, and *** *p* < 0.001.

The antioxidant effect of FSLE was further evaluated by measuring SOD activity and MDA content in mouse liver tissues. As shown in Figure 3A,B, SOD activity and MDA levels in the NC group were 11.75 ± 0.97 U/mg protein and 31.23 ± 4.34 nmol/mg protein, respectively. Compared with the NC group, SOD activity in the Fer group significantly increased to 16.75 ± 1.50 U/mg protein. Concomitantly, MDA content significantly decreased to 15.38 ± 2.46 nmol/mg protein, indicating an enhancement of hepatic antioxidant capacity. The Unfer group showed modest improvements in SOD and MDA levels, but the effects were less pronounced than those in the Fer group. The PC group also showed modest improvements.

In addition, the mRNA expression levels of antioxidant-related genes HO-1 and Nrf2 were assessed in mouse liver tissues (Figure 3C,D). The Fer group displayed significant upregulation of both HO-1 and Nrf2 mRNA, consistent with the observed increase in SOD activity and decrease in MDA levels. Unfer and PC groups also showed moderate increases, but these were lower than those observed in the Fer group. Furthermore, protein-level analysis revealed that Nrf2 expression in tumor tissues was significantly downregulated in the Fer group compared with the NC group (Figure 3E,F).

#### 3.2.3. Safety of FSLE

**Figure 4:**
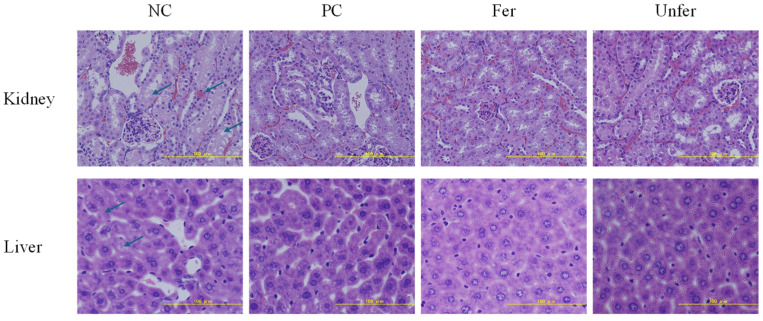
Effects of FSLE on the histological morphology of liver and kidney tissues in mice. Scale bar = 100 μm.

To further evaluate the in vivo safety of FSLE, liver and kidney tissues from each group were subjected to hematoxylin and eosin (HE) staining (Figure 4). In the NC group, renal tissues exhibited mild tubular epithelial swelling with pale cytoplasm, partial narrowing of tubular lumina, and marked congestion in the interstitial capillaries. In contrast, the kidney structures in the Fer group were well-preserved, with intact glomeruli, orderly arranged tubules, and normal epithelial cell morphology; interstitial congestion was notably reduced. Similarly, liver tissues from the Fer group displayed essentially normal histological architecture, with regularly arranged hepatic cords and hepatocytes exhibiting normal morphology, without apparent necrosis or inflammatory infiltration. In comparison, slight vacuolation was observed between hepatocytes in the NC and PC groups.

### 3.3. FSLE Inhibits Inflammation and Enhances Anti-Tumor Immunity

**Figure 5:**
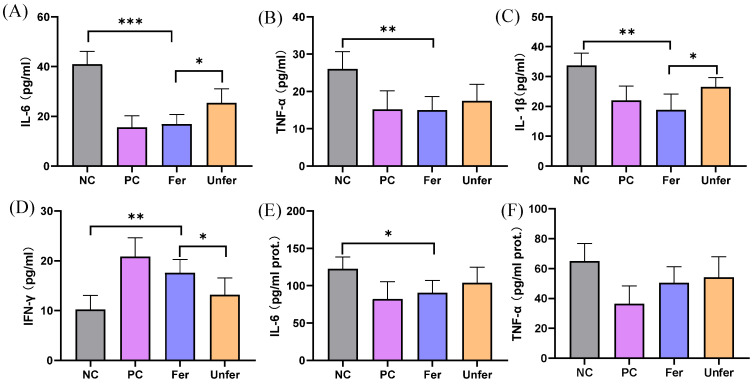
Serum and tumor tissue levels of inflammatory and immune cytokines in tumor-bearing mice treated with FSLE. (**A**) IL-1β in serum; (**B**) TNF-α in serum; (**C**) IL-6 in serum; (**D**) IFN-γ in serum; (**E**) TNF-α in tumor tissues; (**F**) IL-6 in tumor tissues. Data are presented as mean ± SD. * *p* < 0.05, ** *p* < 0.01, and *** *p* < 0.001.

Serum levels of TNF-α, IL-6, IL-1β, and IFN-γ in mice are shown in Figure 5A–D. In the NC group, the levels of TNF-α, IL-6, and IL-1β were 26.0 ± 4.7, 40.9 ± 5.2, and 33.7 ± 4.1 pg/mL, respectively. In contrast, these pro-inflammatory cytokines were significantly decreased in the Fer group to 15.1 ± 3.6, 16.9 ± 3.8, and 18.8 ± 5.3 pg/mL, which was stronger than that in the Unfer group (17.5 ± 4.5, 25.4 ± 5.6, and 26.5 ± 3.2 pg/mL). Moreover, IFN-γ levels in the Fer group were markedly increased to 17.6 ± 2.7 pg/mL, compared with 10.2 ± 2.8 pg/mL in the NC group. The Unfer group also exhibited a moderate increase in IFN-γ (13.2 ± 3.4 pg/mL).

In addition to serum cytokines, the levels of IL-6 and TNF-α in tumor tissues were also modulated by FSLE treatment (Figure 5E,F). In the NC group, IL-6 levels reached 122.8 ± 15.5 pg/mg protein, whereas it was reduced to 88.1 ± 16.7 pg/mg protein in the Fer group. The Unfer group showed a moderate decrease (103.8 ± 21.1 pg/mg protein), and the reduction observed in the Fer group was comparable to that of the PC group (82.1 ± 23.2 pg/mg protein). Similarly, the TNF-α levels tended to decrease, although the reduction in the Fer group did not reach statistical significance. TNF-α levels were highest in the NC group (65.1 ± 11.6 pg/mg protein) and were decreased in both the PC (36.5 ± 11.8 pg/mg protein) and Fer groups (51.5 ± 12.4 pg/mg protein). The Unfer group also showed a moderate reduction (54.2 ± 13.9 pg/mg protein).

### 3.4. FSLE Promotes Tumor Cell Apoptosis

**Figure 6:**
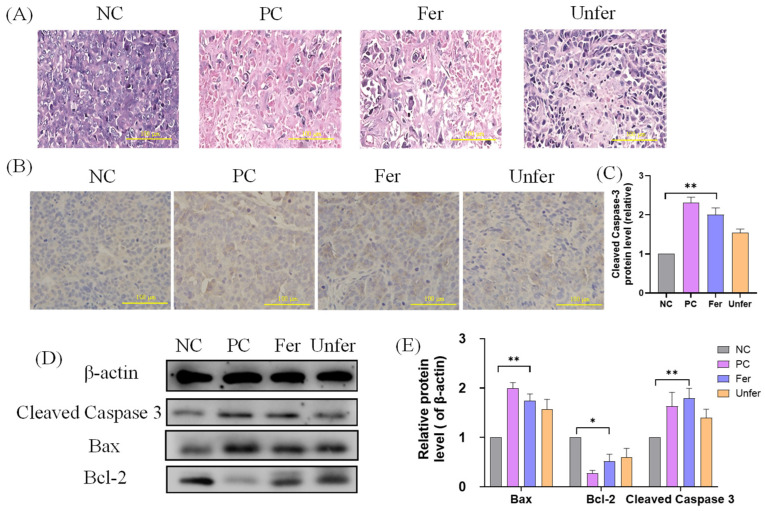
FSLE induces apoptosis in tumor tissues. (**A**) HE staining of tumor sections from each group (scale bar = 100 μm). (**B**) IHC detection of cleaved caspase-3 protein expression in tumor tissues (scale bar = 100 μm). (**C**) Quantification of cleaved caspase-3 protein staining intensity in tumor tissues. Data are presented as relative IOD values normalized to the NC group. (**D**) Western blot analysis of cleaved caspase-3, Bax, and Bcl-2 protein expression in tumor tissues. (**E**) Relative protein levels of Bax, Bcl-2, and cleaved caspase-3 normalized to β-actin. Data are presented as mean ± SD. * *p* < 0.05 and ** *p* < 0.01.

To further elucidate the mechanisms by which FSLE inhibits tumor growth, we examined histopathological features and apoptosis-related markers in tumor tissues. HE staining (Figure 6A) revealed that tumors in the NC group exhibited dense, well-defined cellular structures with minimal degeneration, whereas FSLE-treated tumors displayed extensive cellular disruption, irregular nuclear morphology, and pronounced necrotic areas. In addition, IHC staining (Figure 6B) showed a marked increase in cleaved caspase-3–positive cells in the Fer group, whereas the NC group exhibited only sparse staining and the Unfer group showed a moderate increase. Quantitative analysis (Figure 6C) confirmed that cleaved caspase-3 expression was lowest in the NC group, moderately elevated in the Unfer group, and markedly increased in both the PC and Fer groups.

Western blot results (Figure 6D,E) demonstrated that FSLE markedly increased the levels of cleaved caspase-3 and the pro-apoptotic protein Bax while decreasing the anti-apoptotic protein Bcl-2, leading to a significant elevation of the Bax/Bcl-2 ratio. Consistent with the IHC results, the Fer and PC groups showed higher levels of apoptosis-related proteins compared with the NC group.

## 4. Discussion

In this study, we systematically evaluated the anti-pancreatic cancer effects of FSLE using the PANC-1 xenograft mouse model. FSLE significantly delayed tumor growth and reduced terminal tumor weight while exhibiting a favorable overall toxicity profile, suggesting its potential as a candidate low-toxicity natural adjuvant. These findings align with recent interest in natural products for cancer prevention and treatment [20,21]. Previous analytical characterization of the fermented stevia extract demonstrated that fermentation markedly increased the level of chlorogenic acid methyl ester, which represents the most clearly defined compositional change induced by fermentation. The fermentation-associated increase in chlorogenic acid methyl ester, together with other potential but uncharacterized compositional changes, may be associated with the stronger anti-tumor activity observed for FSLE in the present study. Notably, unlike conventional chemotherapeutics, fermented products often exert anti-tumor effects via context-dependent, multi-target synergistic mechanisms: they can directly inhibit tumor cell proliferation and indirectly modulate host immune and metabolic environments, which may mitigate drug resistance and adverse effects [22].

At the molecular level, FSLE may exert anti-tumor activity through converging anti-inflammatory and redox-modulatory mechanisms. In our study, FSLE significantly reduced both circulating and intratumoral pro-inflammatory cytokines, including IL-6 and TNF-α. However, while FSLE markedly decreased the intratumoral IL-6 levels, the reduction in intratumoral TNF-α did not reach statistical significance. This observation is biologically relevant because chronic inflammation—and in particular sustained activation of the IL-6/JAK/STAT3 axis—is a well-established driver of pancreatic carcinogenesis, promoting tumor cell proliferation, survival, epithelial–mesenchymal transition, invasion, and immune evasion [23,24]. Therefore, the pronounced inhibition of IL-6, especially within the tumor microenvironment, is compatible with the possibility that FSLE may be associated with modulation of IL-6-related inflammatory signaling, thereby alleviating pro-survival and immunosuppressive cues associated with pancreatic tumor progression, while the modest intratumoral decrease in TNF-α may reflect a weaker or more variable local response. Because STAT3 activation and phosphorylation were not directly examined in the present study, these pathway-level interpretations should be regarded as biologically plausible but inferential, based primarily on cytokine expression patterns observed in vivo rather than direct mechanistic verification.

Two complementary mechanisms may underlie the observed anti-inflammatory effects. First, phenolic metabolites enriched or biotransformed during LAB fermentation—particularly chlorogenic acid and related caffeoylquinic derivatives—have been reported to modulate canonical inflammatory signaling pathways, including NF-κB and JAK/STAT, in diverse cellular systems, leading to the reduced expression of pro-inflammatory cytokines such as IL-6 and TNF-α [25,26]. These well-documented molecular activities provide a mechanistic rationale for interpreting the anti-inflammatory outcomes observed in the present in vivo study. Second, metabolites and bioactive factors derived from *L. plantarum*, including cell-free supernatants, exopolysaccharides, and fermentation-associated small molecules, have been shown to attenuate host inflammatory responses [27,28]. Previous in vitro and in vivo studies demonstrate that *L. plantarum* strains can suppress NF-κB activation, reduce IL-6 and in certain contexts TNF-α expression, and modulate cytokine profiles toward a less pro-inflammatory state [29,30]. Collectively, these findings provide a coherent biological basis for the anti-inflammatory effects observed in the current in vivo model and support the possibility that synergistic interactions between plant-derived phenolics and probiotic-derived metabolites contribute to the immunomodulatory properties of FSLE.

Antioxidant and stress-response regulation represents an additional mechanistic component underlying FSLE’s activity. In this study, FSLE upregulated hepatic Nrf2 and HO-1 mRNA expression and was associated with increased SOD activity, reduced MDA levels, and histological evidence of hepatic and renal protection by HE staining. These results suggest that FSLE enhances systemic antioxidant capacity and attenuates oxidative injury. Phenolic acids and certain bacterial metabolites may (i) directly scavenge reactive oxygen species (ROS), (ii) enhance endogenous antioxidant defenses (SOD, catalase, and the GSH system), and (iii) modulate redox sensors such as Nrf2 [31]. Controlled attenuation of oxidative stress may limit DNA damage and suppress pro-tumor inflammation in early tumorigenesis; in established tumors, altering ROS homeostasis can impair redox-dependent survival signaling or sensitize cancer cells to apoptosis [32]. Importantly, Nrf2 signaling is context-dependent: while transient Nrf2 activation protects normal tissues from oxidative stress, persistent or aberrant activation can support metabolic adaptation and drug resistance in certain cancers [33,34]. Consistent with this dual role, FSLE promoted Nrf2 activation in the liver while reducing Nrf2 protein levels in tumor tissues, suggesting a tissue-selective regulatory pattern rather than uniform pathway activation. Because these interpretations are based primarily on expression-level analyses rather than targeted pathway manipulation, the upstream regulatory signals and functional consequences of Nrf2/HO-1 modulation remain areas for future mechanistic investigation. Speculatively, this tumor-specific reduction in Nrf2 may also be indirectly associated with concurrent modulation of IL-6–related signaling, which has been reported to intersect with redox-responsive pathways [35]. Such differential regulation may strengthen antioxidant defenses in normal organs while avoiding excessive Nrf2 activation within the tumor microenvironment.

Based on coordinated cytokine and redox changes observed in vivo, FSLE could potentially influence innate immune components within the tumor milieu. Evidence from multiple studies suggests that chlorogenic acid and related caffeoylquinic derivatives and plant-derived polysaccharides can promote macrophage repolarization from an M2-like (tumor-promoting) to an M1-like (tumor-suppressive) phenotype [36] while enhancing CD8^+^ T-cell activity and anti-tumor immunity [37]. Given the known links between redox regulation, cytokine signaling, and immune cell activation, the coordinated cytokine reduction and antioxidant modulation observed here are compatible with a shift toward a less immunosuppressive tumor milieu. Because immune cell populations were not directly quantified in the present study, these immune-related interpretations should be considered biologically plausible but inferential and warrant dedicated mechanistic validation in future studies. Such interactions may collectively contribute to a regulatory network that is associated with restricted tumor growth, enhanced apoptotic susceptibility, and the overall anti-tumor efficacy of FSLE.

The IHC analysis of tumor tissues demonstrated that FSLE treatment increased the abundance of cleaved caspase-3-positive cells. This histological evidence confirms apoptosis induction at the tissue level and directly corroborates the biochemical findings, consistent with the activation of caspase-dependent apoptotic processes. Consistently, Western blot analysis showed that FSLE markedly increased the expression of pro-apoptotic proteins Bax and cleaved caspase-3 while decreasing the anti-apoptotic protein Bcl-2, suggesting engagement of mitochondria-associated apoptotic signaling. Apoptosis is a tightly regulated form of programmed cell death that maintains cellular homeostasis [38], and its induction is a major therapeutic objective because it enables the selective elimination of malignant cells [39]. The Bcl-2 protein family critically regulates mitochondrial apoptosis by controlling cytochrome c release and the subsequent activation of caspases [29,30], while caspase-3 functions as a key executioner in this cascade [40]. Given that pancreatic tumors frequently exhibit anti-apoptotic phenotypes [41], the observed Bax/Bcl-2 shift and enhanced caspase-3 activation are consistent with a biologically meaningful pro-apoptotic effect of FSLE in vivo. Moreover, previous studies demonstrate that LAB fermentation improves the bioavailability of polyphenolic compounds, enhancing their effects on mitochondrial membrane potential and apoptotic signaling [40,41], with similar mechanisms reported for fermented ginseng, ginsenosides, and soy isoflavones [42,43,44]. These mechanistic parallels provide a rationale for the future investigation of FSLE as a potential apoptosis-modulating and chemosensitizing adjunct in pancreatic cancer treatment.

In summary, this study provides in vivo experimental evidence supporting the anti-tumor efficacy of FSLE against pancreatic cancer. The observed effects are consistent with coordinated multi-target biological responses, including attenuation of the inflammatory tumor microenvironment, engagement of apoptosis-related signaling pathways, and enhancement of antioxidant defenses. Importantly, FSLE exhibited no apparent toxicity in the present animal experiments, highlighting its potential as a safe and low-toxicity natural adjuvant for cancer therapy. Rather than acting through a single dominant pathway, FSLE appears to exert coordinated regulatory effects across the inflammatory, oxidative, and apoptotic networks. These findings broaden the scope of research on plant-derived fermentation products in oncology and provide a preliminary experimental framework to support future investigations into the development of nutritional adjuvants in cancer management. Several limitations of this study should be acknowledged. First, the sample size of four mice per group, while sufficient to detect the reported significant differences, was relatively small and may limit the statistical power and generalizability of the findings. Second, the proposed mechanisms, particularly the differential regulation of Nrf2, are preliminary. We did not investigate upstream regulators such as Keap1 or p62, nor did we examine Nrf2 nuclear translocation. Third, the lack of transcriptomic or proteomic profiling precludes a comprehensive understanding of the signaling networks modulated by FSLE. Future studies with larger cohorts and multi-omics approaches are warranted to validate and extend these observations. In addition, the identification of key bioactive metabolites and the evaluation of potential combination strategies with conventional chemotherapeutic agents represent important future research directions. Comprehensive cellular, pharmacological, and multi-omics investigations will be valuable for further elucidating the molecular basis of FSLE-mediated anti-tumor activity.

## 5. Conclusions

This study systematically evaluated the anti-tumor effects of FSLE in a PANC-1 xenograft mouse model. FSLE significantly inhibited tumor growth and was associated with reduced tumor-associated inflammation, increased apoptotic signaling, enhanced systemic antioxidant responses, and improved hepatic and renal histopathology. Mechanistically, the observed anti-tumor activity is consistent with the involvement of mitochondria-mediated apoptotic signaling, as evidenced by modulation of the Bax/Bcl-2 axis and cleaved caspase-3 activation, activation of hepatic Nrf2/HO-1 antioxidant signaling, and suppression of pro-inflammatory cytokines, particularly IL-6. Notably, Nrf2 protein expression was reduced in tumor tissues, suggesting a tissue-selective regulatory pattern that may enhance systemic antioxidant protection while potentially limiting pro-survival Nrf2 signaling within the tumor microenvironment. Importantly, no overt toxicity was observed in the present animal study, including the absence of adverse hepatic or renal histopathological alterations, supporting the safety profile of the fermented product as a potential natural adjuvant for cancer therapy. These findings provide preliminary experimental and theoretical support for the further development and investigation of natural product-based adjunct strategies against pancreatic cancer.

## Data Availability

All data supporting the findings of this study are available within the manuscript.

## References

[1] Qian Y., Gong Y., Fan Z., Luo G., Huang Q., Deng S., Cheng H., Jin K., Ni Q., Yu X. (2020). Molecular alterations and targeted therapy in pancreatic ductal adenocarcinoma. J. Hematol. Oncol..

[2] Bray F., Laversanne M., Sung H., Ferlay J., Siegel R.L., Soerjomataram I., Jemal A. (2024). Global cancer statistics 2022: GLOBOCAN estimates of incidence and mortality worldwide for 36 cancers in 185 countries. CA Cancer J. Clin..

[3] Stoop T.F., Javed A.A., Oba A., Koerkamp B.G., Seufferlein T., Wilmink J.W., Besselink M.G. (2025). Pancreatic cancer. Lancet.

[4] Dreyer S.B., Beer P., Hingorani S.R., Biankin A.V. (2025). Improving outcomes of patients with pancreatic cancer. Nat. Rev. Clin. Oncol..

[5] Xing P., Zhong Y., Cui X., Liu Z., Wu X. (2023). Natural products in digestive tract tumors metabolism: Functional and application prospects. Pharmacol. Res..

[6] Wang Y., Wang F., Liu W., Geng Y., Shi Y., Tian Y., Zhang B., Luo Y., Sun X. (2024). New drug discovery and development from natural products: Advances and strategies. Pharmacol. Ther..

[7] Peteliuk V., Rybchuk L., Bayliak M., Storey K.B., Lushchak O. (2021). Natural sweetener Stevia rebaudiana: Functionalities, health benefits and potential risks. EXCLI J..

[8] Iatridis N., Kougioumtzi A., Vlataki K., Papadaki S., Magklara A. (2022). Anti-cancer properties of *stevia rebaudiana*; more than a sweetener. Molecules.

[9] Kasti A.N., Nikolaki M.D., Synodinou K.D., Katsas K.N., Petsis K., Lambrinou S., Pyrousis I.A., Triantafyllou K. (2022). The effects of stevia consumption on gut bacteria: Friend or foe?. Microorganisms.

[10] Martinez-Rojo E., Carino-Cortes R., Berumen L.C., Garcia-Alcocer G., Escobar-Cabrera J. (2020). Stevia eupatoria and stevia pilosa extracts inhibit the proliferation and migration of prostate cancer cells. Medicina.

[11] Chai L.J., Lan T., Cheng Z., Zhang J., Deng Y., Wang Y., Li Y., Wang F., Piao M. (2024). *Stevia rebaudiana* leaves fermented by *Lactobacillus plantarum* exhibit resistance to microorganisms and cancer cell lines in vitro: A potential sausage preservative. Food Chem..

[12] Guo R., Guo S., Gao X., Wang H., Hu W., Duan R., Dong T.T.X., Tsim K.W.K. (2020). Fermentation of *Danggui Buxue Tang*, an ancient Chinese herbal mixture, together with *Lactobacillus plantarum* enhances the anti-diabetic functions of herbal product. Chin. Med..

[13] Ricci A., Cirlini M., Calani L., Bernini V., Neviani E., Del Rio D., Galaverna G., Lazzi C. (2019). In vitro metabolism of elderberry juice polyphenols by lactic acid bacteria. Food Chem..

[14] Shakya S., Danshiitsoodol N., Noda M., Sugiyama M. (2023). Role of phenolic acid metabolism in enhancing bioactivity of mentha extract fermented with plant-derived *Lactobacillus plantarum* SN13T. Probiotics Antimicrob. Proteins.

[15] Fusco V., Fanelli F., Chieffi D. (2022). Authenticity of probiotic foods and dietary supplements: A pivotal issue to address. Crit. Rev. Food Sci. Nutr..

[16] Zhu H., Guo L., Yu D., Du X. (2022). New insights into immunomodulatory properties of lactic acid bacteria fermented herbal medicines. Front. Microbiol..

[17] Eweys A.S., Zhao Y.S., Darwesh O.M. (2022). Improving the antioxidant and anticancer potential of Cinnamomum cassia via fermentation with *Lactobacillus plantarum*. Biotechnol. Rep..

[18] Zhang R., Danshiitsoodol N., Noda M., Yonezawa S., Kanno K., Sugiyama M. (2025). Stevia Leaf Extract Fermented with Plant-Derived *Lactobacillus plantarum* SN13T Displays Anticancer Activity to Pancreatic Cancer PANC-1 Cell Line. Int. J. Mol. Sci..

[19] Ma Q., Noda M., Danshiitsoodol N., Sugiyama M. (2023). Fermented Stevia Improves Alcohol Poisoning Symptoms Associated with Changes in Mouse Gut Microbiota. Nutrients.

[20] Newman D.J., Cragg G.M. (2020). Natural Products as Sources of New Drugs over the Nearly Four Decades from 01/1981 to 09/2019. J. Nat. Prod..

[21] Zarins-Tutt J.S., Barberi T.T., Gao H., Mearns-Spragg A., Zhang L., Newman D.J., Goss R.J. (2016). Prospecting for new bacterial metabolites: A glossary of approaches for inducing, activating and upregulating the biosynthesis of bacterial cryptic or silent natural products. Nat. Prod. Rep..

[22] Atanasov A.G., Zotchev S.B., Dirsch V.M., Supuran C.T., the International Natural Product Sciences Taskforce (2021). Natural products in drug discovery: Advances and opportunities. Nat. Rev. Drug. Discov..

[23] Lesina M., Kurkowski M.U., Ludes K., Rose-John S., Treiber M., Kloppel G., Yoshimura A., Reindl W., Sipos B., Akira S. (2011). Stat3/Socs3 activation by IL-6 transsignaling promotes progression of pancreatic intraepithelial neoplasia and development of pancreatic cancer. Cancer Cell..

[24] Johnson D.E., O’Keefe R.A., Grandis J.R. (2018). Targeting the IL-6/JAK/STAT3 signalling axis in cancer. Nat. Rev. Clin. Oncol..

[25] Huang J., Xie M., He L., Song X., Cao T. (2023). Chlorogenic acid: A review on its mechanisms of anti-inflammation, disease treatment, and related delivery systems. Front. Pharmacol..

[26] Zhang L., Fan Y., Su H., Wu L., Huang Y., Zhao L., Han B., Shu G., Xiang M., Yang J.M. (2018). Chlorogenic acid methyl ester exerts strong anti-inflammatory effects via inhibiting the COX-2/NLRP3/NF-kappaB pathway. Food Funct..

[27] Yang S.Y., Chae S.A., Bang W.Y., Lee M., Ban O.H., Kim S.J., Jung Y.H., Yang J. (2021). Anti-inflammatory potential of *Lactiplantibacillus plantarum* IDCC 3501 and its safety evaluation. Braz. J. Microbiol..

[28] Zhao W., Peng C., Sakandar H.A., Kwok L.Y., Zhang W. (2021). Meta-Analysis: Randomized Trials of *Lactobacillus plantarum* on Immune Regulation Over the Last Decades. Front. Immunol..

[29] Chen G., Qian W., Li J., Xu Y., Chen K. (2015). Exopolysaccharide of Antarctic bacterium Pseudoaltermonas sp. S-5 induces apoptosis in K562 cells. Carbohydr. Polym..

[30] Sungur T., Aslim B., Karaaslan C., Aktas B. (2017). Impact of Exopolysaccharides (EPSs) of *Lactobacillus gasseri* strains isolated from human vagina on cervical tumor cells (HeLa). Anaerobe.

[31] Cheng J.-C., Dai F., Zhou B., Yang L., Liu Z.-L. (2007). Antioxidant activity of hydroxycinnamic acid derivatives in human low density lipoprotein: Mechanism and structure–activity relationship. Food Chem..

[32] Perillo B., Di Donato M., Pezone A., Di Zazzo E., Giovannelli P., Galasso G., Castoria G., Migliaccio A. (2020). ROS in cancer therapy: The bright side of the moon. Exp. Mol. Med..

[33] Trachootham D., Alexandre J., Huang P. (2009). Targeting cancer cells by ROS-mediated mechanisms: A radical therapeutic approach?. Nat. Rev. Drug. Discov..

[34] DeNicola G.M., Karreth F.A., Humpton T.J., Gopinathan A., Wei C., Frese K., Mangal D., Yu K.H., Yeo C.J., Calhoun E.S. (2011). Oncogene-induced Nrf2 transcription promotes ROS detoxification and tumorigenesis. Nature.

[35] Wruck C.J., Streetz K., Pavic G., Gotz M.E., Tohidnezhad M., Brandenburg L.O., Varoga D., Eickelberg O., Herdegen T., Trautwein C. (2011). Nrf2 induces interleukin-6 (IL-6) expression via an antioxidant response element within the IL-6 promoter. J. Biol. Chem..

[36] Xue N., Zhou Q., Ji M., Jin J., Lai F., Chen J., Zhang M., Jia J., Yang H., Zhang J. (2017). Chlorogenic acid inhibits glioblastoma growth through repolarizating macrophage from M2 to M1 phenotype. Sci. Rep..

[37] Wang S., Hu Q., Chang Z., Liu Y., Gao Y., Luo X., Zhou L., Chen Y., Cui Y., Wang Z. (2023). Moringa oleifera leaf polysaccharides exert anti-lung cancer effects upon targeting TLR4 to reverse the tumor-associated macrophage phenotype and promote T-cell infiltration. Food Funct..

[38] Mohammad R.M., Muqbil I., Lowe L., Yedjou C., Hsu H.Y., Lin L.T., Siegelin M.D., Fimognari C., Kumar N.B., Dou Q.P. (2015). Broad targeting of resistance to apoptosis in cancer. Semin. Cancer Biol..

[39] Evan G.I., Vousden K.H. (2001). Proliferation, cell cycle and apoptosis in cancer. Nature.

[40] Van Opdenbosch N., Lamkanfi M. (2019). Caspases in Cell Death, Inflammation, and Disease. Immunity.

[41] Cory S., Adams J.M. (2002). The Bcl2 family: Regulators of the cellular life-or-death switch. Nat. Rev. Cancer.

[42] Xu H.Y., Li Q.C., Zhou W.J., Zhang H.B., Chen Z.X., Peng N., Gong S.Y., Liu B., Zeng F. (2023). Anti-Oxidative and Anti-Aging Effects of Probiotic Fermented Ginseng by Modulating Gut Microbiota and Metabolites in Caenorhabditis elegans. Plant Foods Hum. Nutr..

[43] Marazza J.A., Garro M.S., de Giori G.S. (2009). Aglycone production by *Lactobacillus rhamnosus* CRL981 during soymilk fermentation. Food Microbiol..

[44] Wang H., Zheng Y., Sun Q., Zhang Z., Zhao M., Peng C., Shi S. (2021). Ginsenosides emerging as both bifunctional drugs and nanocarriers for enhanced antitumor therapies. J. Nanobiotechnol..

